# Dramatic response to trastuzumab-deruxtecan in metastatic HER2-overexpressing vulvar extramammary Paget's disease: A case report

**DOI:** 10.1016/j.gore.2026.102213

**Published:** 2026-09-17

**Authors:** Myrtille Thomas, Mojgan Devouassoux-Shisheboran, Jonathan Lopez, Pierre Descargues, Witold Gertych, Ionela Caraivan, Benoit You, Julien Péron

**Affiliations:** a Oncology department, CHU de Lyon SUD, Hospices Civils de Lyon, France; b Pathology department, CHU de Lyon SUD, Hospices Civils de Lyon, France; c Department of Biochemistry and Molecular Biology, CHU de Lyon SUD, Hospices Civils de Lyon, France; d Department of Gynecological Surgery and Oncology, Obstetrics, CHU de Lyon SUD, Hospices Civils de Lyon, France; e Department of Gynecological Surgery and Oncology, Obstetrics, Lausanne University Hospital, Switzerland; f Radiotherapy department, CHU de Lyon SUD, Hospices Civils de Lyon, France; g Medical oncology, EPSILYON, Lyon University Hospital, Univ Lyon 1, CICLY, Lyon, France; GINECO, Paris, France

**Keywords:** Vulvar EMPD, HER2 overexpression, Trastuzumab-deruxtecan, Metastatic disease

## Abstract

**Background:**

Extramammary Paget's disease (EMPD) is a rare cutaneous in situ adenocarcinoma sometimes associated with invasive malignancy that may evolve to metastatic disease. HER2 alterations have been reported in aggressive cases and may be targetable.

**Case presentation:**

We report a patient with metastatic vulvar EMPD with HER2 3+ score by immunohistochemistry (IHC) and *ERBB2* amplification who progressed after local therapies, surgery, radiotherapy and trastuzumab ± paclitaxel. Off-label trastuzumab-deruxtecan (T-DXd) was started as second-line systemic therapy with marked extracranial and intracranial responses and acceptable toxicity.

**Conclusion:**

This case supports consideration of HER2-directed antibody-drug conjugates in HER2-positive EMPD when standard options are exhausted.

## Highlights

• Metastatic vulvar EMPD are usually associated with HER2 overexpression.
• Paclitaxel and trastuzumab combination was associated with a complete and durable extracranial response.
• 2nd line Trastuzumab-deruxtecan (T-DXd) was associated with a durable intracranial response.

## 1 Introduction

Extramammary Paget's disease (EMPD) is a rare neoplasm that usually develops in the apocrine gland-bearing areas of older adults (Ishizuki and Nakamura, 2021). While primary EMPD arises as intraepithelial neoplasm of the epidermis, secondary EMPD develops from the extension of an underlying internal carcinoma.

EMPD mostly presents as an intraepithelial adenocarcinoma with favourable prognosis, however invasive lesions can develop in up to 25% of cases, with the potential to evolve to metastatic diseases (Cai et al., 2013). Half of metastatic cases harbor HER2 overexpression associated with *ERBB2* amplification (Fukuda and Funakoshi, 2018).

Local therapy such as topical treatment, surgery or radiotherapy, is standard when feasible (Luyten et al., 2014; Nasioudis et al., 2020). Systemic options for inoperable/metastatic diseases are not standardized, and the use of variable chemotherapy regimen have been reported (Kibbi et al., 2022; Pérez et al., 2023). Recent success of HER2-targeted agents (trastuzumab, trastuzumab-emtansine, pertuzumab combinations and trastuzumab-deruxtecan) in other tumors provides rationale for their use in HER2-positive EMPD (Swain et al., 2020; Cortés et al., 2022; Li et al., 2022; Shitara et al., 2025).

## 2 Case presentation

Our patient, 78 years old female, with no relevant medical history or medication, presented with an erosive vulvar lesion that had appeared a year ago and had previously failed topical antifungals and topical antibiotics therapy. On examination, the erythematous lesion measured approximately 4 cm in diameter and extended to the right labium majus, the perianal region and the upper right thigh. Biopsy demonstrated vulvar extramammary Paget's disease (EMPD), with a consistent immunohistochemical profile (CK7-positive, CK20-negative, uroplakin III-negative). Radiological workup showed no involvement of the vagina, urethra or rectum, no pathological lymph node nor deep local extension.

The patient was initially treated with topical imiquimod, topical betamethasone dipropionate and laser vaporization, with a partial response lasting one year. On recurrence, surgery was recommended. She underwent two successive partial vulvectomies, both of which showed Paget's disease with a dermal invasive component. The first surgical procedure had margins free of invasive carcinoma but involved by carcinoma in situ (CIS) on all of them; the second resection had likewise no invasive carcinoma and persistent CIS on all the margins.

Three months after the second surgical procedure, multiple right inguinal nodes were identified. A PET-scan confirmed a hypermetabolism in this area. A right inguinal lymph node dissection was performed finding 6 N+/12 with a pathological examination consistent with Paget's adenocarcinoma, HER2 immunohistochemistry (IHC) was 3+ score and molecular analysis found *ERBB2* amplification, and *CDK12* co-amplification, heterozygous loss of *ARID1A*; proficient MMR.

The patient received adjuvant radiation therapy of the vulva (60 Gy in 30 fractions) then radiation of bilateral inguinal and pelvic node area (45 Gy then a boost on right inguinal area up to 50 Gy).

Eight months after the end of the radiation therapy, CT-scan showed two left upper-lobe pulmonary nodules and suspicious interaortocaval nodes. Clinical examination identified recurrent vulvar adenocarcinoma. No recurrence on clinical examination nor imaging was found in the treated groin. A pulmonary biopsy was performed and confirmed the metastatic relapse of the invasive component of her EMPD. Given metastatic disease, first-line systemic therapy was initiated with weekly paclitaxel associated with trastuzumab. After 3 cycles there was a partial response of the pulmonary lesions and vulvar disease. Paclitaxel was reduced by 25% for the fourth cycle due to grade 2 sensory neuropathy and discontinued at the end of the fourth cycle because of persistent symptoms. Trastuzumab was maintained for a total of 10 months.

While there was no vulvar or pulmonary progression, the patient was admitted to the hospital for confusion. A brain MRI revealed multiple brain metastases. Given the exclusively cerebral progression on trastuzumab, off-label trastuzumab-deruxtecan (T-DXd) was started (initial reduced dose of 4.4 mg/kg given the age and clinical setting of the patient with primary G-CSF prophylaxis; second cycle dose lowered to 3.2 mg/kg due to grade 2 asthenia). An acute atrial fibrillation episode with transient troponin rise occurred prior to the second infusion and resolved with beta-blocker and anticoagulation without echocardiographic dysfunction.

## 3 Response and follow-up

Restaging imaging after four cycles demonstrated a marked partial response of the intracranial disease. Follow-up imaging after eight cycles confirmed a persistent complete metabolic response of extracranial (vulvar, pulmonary and lymph nodes) lesions on the PET-scan and a deepening partial response of the intracranial disease on the MRI. The assessment after 13 cycles, documented sustained extracranial disease control but multifocal progression of brain metastases, prompting whole-brain radiation therapy (37,5Gy in 15 fractions) and resuming T-DXd afterward. The patient has received 18 infusions after fourteen months of treatment. A brain MRI was performed after her 18th cycle, revealing a reduction in size of known brain metastasis without any new secondary brain lesions (Fig. 1). There was no grade 2 or greater toxicity reported since the treatment initiation. Grade 1 asthenia and anorexia is to be noted.

**Fig. 1:**
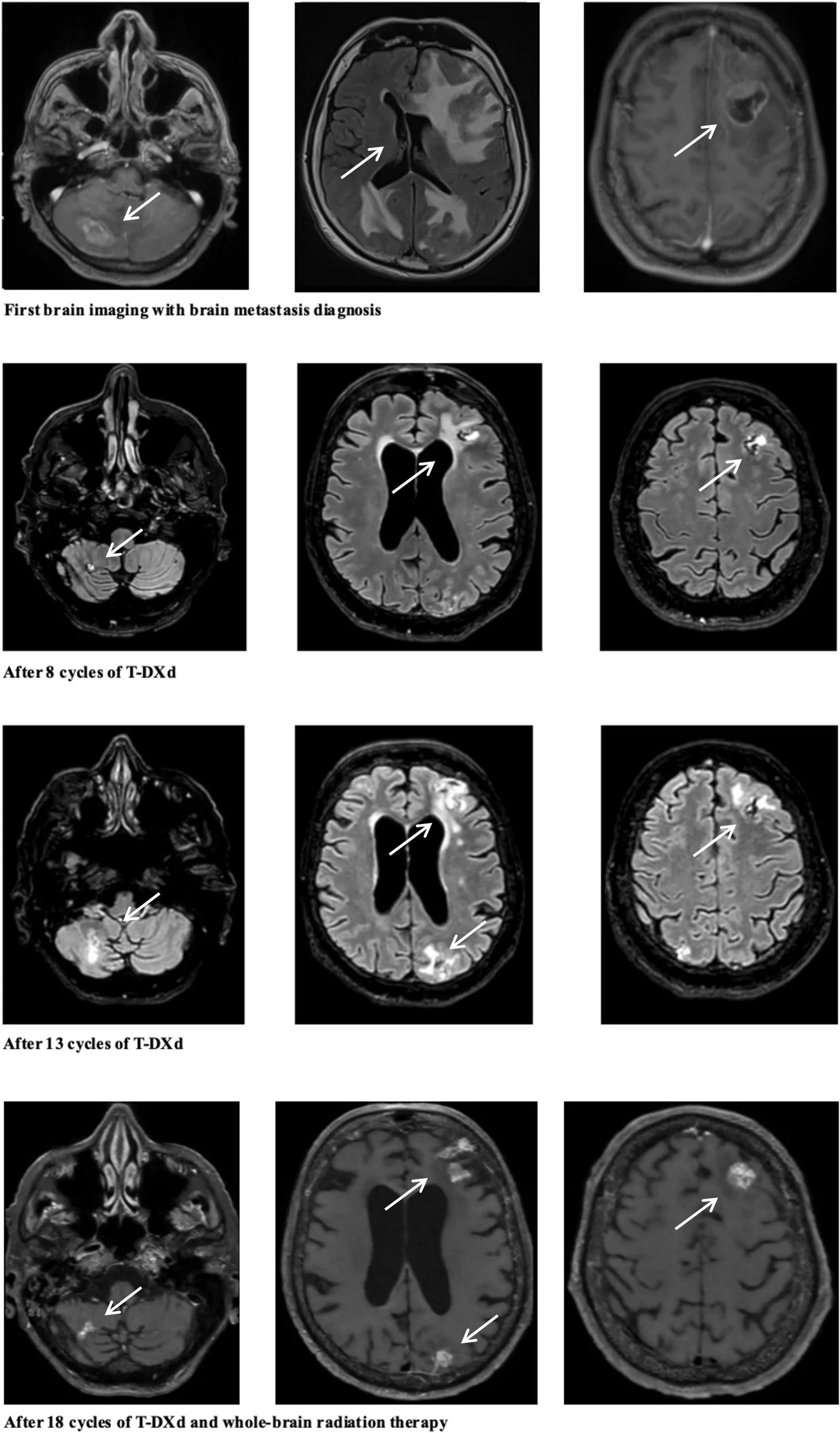
Brain MRI images at diagnosis, 8 cycles, 13 cycles and 18 cycles of T-DXd.

## 4 Discussion

This case illustrates significant activity of T-DXd in brain metastases and extracranial lesions of HER2-amplified metastatic vulvar EMPD with dermal invasion after brain progression on trastuzumab plus paclitaxel. HER2 alterations are reported in a subset of aggressive EMPD and may predict clinical response from HER2-targeted therapies (Tanaka et al., 2013). Prior reports describe responses to trastuzumab, trastuzumab-emtansine, and dual HER2 blockade in EMPD (Karam et al., 2008; Hanawa et al., 2011; Wakabayashi et al., 2012; Bartoletti et al., 2020; Hsieh et al., 2018).

T-DXd has shown high activity across HER2-positive tumors (breast, gastric, lung) and emerging evidence supports its use in other HER2-expressing malignancies including EMPD (Meric-Bernstam et al., 2024; Gorecki et al., 2026). The use of T-DXd has already been validated in the case of brain metastasis in breast cancer, in our case report its activity seems to not be as durable as expected (Harbeck et al., 2024). Challenges in EMPD include rarity, heterogeneous treatment approaches, and limited prospective data. This case report highlights the importance of molecular profiling in rare cancers, including EMPD, to detect *ERBB2* alterations and underscores the need for innovative targeted therapies available for off-label use in this patient population. Safety monitoring for ILD and cardiac events is essential with T-DXd.

This report has several limitations. It describes a single patient, baseline imaging was incomplete, and the treatment was given off-label and at reduced doses, so the response observed here may not be reproductible in other patients. Nonetheless, the case illustrates the value of molecular profiling in rare tumors such as EMPD, where the identification of an *ERBB2* alterations opened access to an effective targeted therapy.

In patients with HER2-overexpressing metastatic EMPD, refractory to earlier HER2-directed therapy especially in cases of a solitary cerebral progression, trastuzumab-deruxtecan can produce a marked and lasting response to both cerebral and extracerebral metastases, with manageable toxicity. Molecular testing for HER2 should be performed on all advanced EMPD to identify candidates who could benefit from HER2-targeted therapies.

## Informed consent and patient

Written informed consent to take part in the study and to publish the article has been obtained from all participants or their legal representatives. The privacy rights of participants have been observed.

## Studies in human

This study was performed in compliance with relevant laws, regulatory frameworks and guidelines where the research took place.

This study was approved by the CHU LYON SUD.

(Approval No. Opinion N° 26–278, CNIL N° 26–5278.)

## Case reports

This research follows the CARE guidelines and the CARE checklist.

## Patient consent/ethics committee

Written informed consent for publication was obtained from the patient— include per journal requirements.

This case report was approved by an ethics committee, Opinion N° 26–278, CNIL N° 26–5278.

## Declaration of generative ai technologies in the writing process

During the preparation of this work, the authors used DeepL and Claude to improve readability and language. The authors reviewed and edited the output as needed and take full responsibility for the content of the published article.

## CRediT authorship contribution statement

**Myrtille Thomas:** Writing – review & editing, Writing – original draft, Methodology, Conceptualization. **Mojgan Devouassoux-Shisheboran:** Writing – review & editing. **Jonathan Lopez:** Writing – review & editing. **Pierre Descargues:** Writing – review & editing. **Witold Gertych:** Writing – review & editing. **Ionela Caraivan:** Writing – review & editing. **Benoit You:** Writing – review & editing. **Julien Péron:** Writing – review & editing, Writing – original draft, Supervision, Conceptualization.

## Declaration of competing interest

The authors declare that they have no known competing financial interests or personal relationships that could have appeared to influence the work reported in this paper.
